# Transplacental transmission of torque teno virus

**DOI:** 10.1186/s12985-017-0762-0

**Published:** 2017-05-08

**Authors:** Elena A. Tyschik, Sophia M. Shcherbakova, Ruslan R. Ibragimov, Denis V. Rebrikov

**Affiliations:** 1 Kulakov Research Center for Obstetrics, Gynecology and Perinatology, 117997 Oparina 4, Moscow, Russia; 2 Pirogov Russian National Research Medical University, 117997 Ostrovityanova 1, Moscow, Russia

**Keywords:** Torque Teno Virus, Transfusion transmitted virus, TTV, Transplacental transmission, Cord blood, Mother-to-child

## Abstract

**Background:**

TTV has been detected in almost every human tissue type or body fluid reaching near 100% prevalence. Several studies report mother-to-child postnatal transmission of TTV in infancy but the risk of transplacental transmission of TTV is still unclear.

**Methods:**

The blood and plasma collected postpartum from 100 mother-child pairs were analyzed using TTV-specific qPCR. Samples were collected from the peripheral vein of the mother and the umbilical cord.

**Results:**

Eighty four percent of pregnant women were TTV positive (median titers: 8 × 10^4^ copies/mL; range: 10^3^ – 3 × 10^7^). The TTV load in plasma was approximately 100 times lower than in whole blood. TTV was not detected in any of cord blood samples.

**Conclusions:**

Our data demonstrate the lack of transplacental transmission of TTV (or effective prenatal inhibition of viral proliferation). The presence of the virus in infants may be associated with mother-to-child transmission through breast feeding or other routes of transmission.

## Background

Torque teno virus (TTV) is a small virus of the Anelloviridae family with a circular ssDNA genome of negative polarity; it was first reported in 1997 in Japanese patients with non-A-G posttransfusion hepatitis [1]. TTV has been detected in almost every human tissue type or body fluid reaching near 100% prevalence [2, 3]; the virus is thought to be transmitted through breast feeding, by fecal-oral, respiratory, or sexual routes, through contaminated water, etc. [4]. Cross-species swine-to-human transmission of TTVs has also been reported [5]. Some data suggest TTV plasma load association with age and gender [6].

Mother-to-child transmission of viral infections can be transplacental, perinatal (through vaginal fluids or blood), or postnatal (through breast feeding or other routes). Transplacental transmission has been demonstrated for many viruses including the rubella virus, cytomegalovirus (CMV), herpes simplex virus (HSV), parvovirus B19 (B19V), varicella-zoster virus (VZV), West Nile virus, measles virus, hepatitis E virus, human immunodeficiency virus (HIV), lymphocytic choriomeningitis virus (LCMV), enteroviruses, adenoviruses, and some other viruses [7].

Several studies report mother-to-child postnatal transmission of TTV in infancy [8–14] but the risk of transplacental transmission of TTV is still unclear. The aim of this study was to quantify TTV in the venous blood and plasma of pregnant women and in the umbilical cord blood and plasma collected postpartum to estimate the possibility of transplacental transmission of TTV.

## Methods

### Ethical approval

The study protocol was reviewed and approved by the Ethics Committee of the Pirogov Russian National Research Medical University (Protocol No.2016/64); the study was conducted in accordance with the Declaration of Helsinki. All participants gave written informed consent. Blood samples obtained from the blood bank were anonymous.

### Blood samples collection

Blood and plasma samples were collected from 100 mother-child pairs over the period from January to November 2016 at Kulakov Research Center for Obstetrics, Gynecology and Perinatology (Moscow, Russia). Mothers’ ages ranged from 20 to 47, with mean age of 31 years. The inclusion criterion was normal singleton pregnancy. The exclusion criteria were as follows: pregnancy complications of any type, premature delivery, multiple pregnancies, and caesarean section. Two separate aliquots of each sample collected postpartum from a peripheral vein of the mother and the umbilical cord were placed into 4 mL EDTA Vacutainers. All samples were stored at +4 °C for less than three hours before they were centrifuged for plasma separation first at 800 g for 5 min and then at 3000 g for 5 min. Then whole blood and plasma were immediately frozen at −80 °C until DNA extraction.

### DNA extraction

DNA was extracted from 200 μl aliquots of thawed whole blood or plasma using a standard commercial silica-sorbent kit for DNA extraction from body fluids (Probe-GS® DNA Extraction Kit, DNA-Technology, Russia). To prevent exogenous contamination, DNA isolation was performed in a separate DNA extraction room (Zone 1). To prevent cross-contamination of the samples, all procedures were carried out in the UV-equipped PCR-box using sterile disposable tubes and aerosol-resistant tips.

### TTV quantification

qPCR was performed using the DTprime Real-Time PCR Cycler (DNA-Technology, Russia) as described in [2], with test sensitivity of about 1000 viral copies per milliliter of blood/plasma. qPCR of the unique human genome fragment was used as DNA extraction control (in a separate PCR tube). To prevent PCR contamination by previous reactions or biological samples, the reactions were combined using aerosol-resistant tips in UV-equipped PCR-box in a separate PCR-preparation room (Zone 2). Also, no electrophoresis of TTV PCR products or other procedures that would require PCR-tube opening were performed in the building. All the negative controls and surface washings were negative.

### Data analysis

qPCR data were analyzed using the DTprime Real-Time PCR Cycler Software v.7.7 (DNA-Technology, Russia). Microsoft Office Excel (Microsoft Corporation, USA) and Statistica 8.0 (StatSoft, USA) were used for statistical analysis.

## Results

**Fig. 1:**
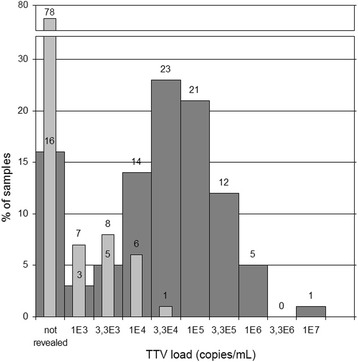
TTV viral load in maternal samples (dark bars represent whole blood samples, light bars – plasma samples). Numbers above each bar represent the number of samples

We have demonstrated that in 84% of pregnant women the TTV viral load is higher than 10^3^ copies per milliliter of whole blood (test sensitivity), with a maximum of 3 × 10^7^ and a median of 8 × 10^4^ viral genomes per 1 mL (see Fig. 1). The TTV load in plasma was approximately 100 times lower than in whole blood (22 samples were positive, with a maximum of 7 × 10^4^ copies/mL) indicating the predominantly cellular localization of the virus.

TTV was not detected in any of cord blood samples. The levels of human genomic DNA were pretty similar in maternal and cord blood samples (an average of 4.1 and 4.2 × 10^8^ copies/mL, respectively).

## Discussion

The revealed presence of TTV in the venous blood of 84% of pregnant women correlates well with the previously published studies on healthy human populations [2, 6]. Because of test sensitivity, only 22% of plasma samples came out positive for TTV. The TTV load in plasma was approximately 100 times lower than in whole blood. The PCR assay we used has sensitivity of about 10^3^ viral genomes per milliliter. Therefore, all samples with a lower viral load were considered negative. In [6] the authors detected TTV in 76% of plasma samples with test sensitivity of 10^2^ copies/mL. Based on the fact that the ratio of genomic DNA in the cells and extracellular fraction is about 1000, we can assume that the virus found in plasma mostly comes from disrupted cells.

This study revealed an approximately 10-fold decline in the median value of the TTV load in whole blood samples compared to the results we obtained from elite athletes in our previous work [2]. This can be explained by changes in the DNA extraction technique, qPCR data analysis algorithm, or initial biological difference of analyzed groups.

## Conclusions

The TTV viral load of more than 1000 copies per 1 mL of whole blood was detected in 84% of pregnant women (with a maximum of 3 × 10^7^ and a median of 8 × 10^4^ copies/mL). The TTV load in plasma was approximately 100 times lower than in whole blood indicating the predominantly intracellular localization of the virus.

Our data demonstrate the lack of transplacental transmission of TTV. The presence of the virus in infants may be associated with mother-to-child transmission through breast feeding or other routes of transmission.

## Abbreviations

PCR: Polymerase chain reaction
qPCR: Quantitative polymerase chain reaction
ssDNA: Single stranded deoxyribonucleic acid
TTV: Torque Teno Virus
UV: Ultraviolet
